# Lectures on Pulmonary Phthisis

**Published:** 1845-04

**Authors:** 


I. Lectures on Pulmonary Phthisis. By *John T. Evans*, M.D. Dublin, 1844. 8vo. pp. 196.

II. Researches on Phthisis. By *P. C. A. Louis*, M.D. Second Edition. Translated by *W. H. Walshe*, M.D. London (Sydenham Society) 1844. 8vo. pp. 566.

III. Synoptical Account of the Effects of Certain Medicines applied to the Treatment of Asthma and Consumption, on the Principle of Endosmosis. By *W. H. Brown*, M.D. 1844. 8vo. pp. 24.

Disheartening as is the fact that notwithstanding the immense amount of labour and talent which has been of late years devoted to the investigation of the phenomena of Phthisis, little or no progress has been made in its therapeutical management, it is gratifying to find that the subject is not abandoned in despair, but still occupies the time and thought of intellects of the highest order. They justly deem it an inconcievable anomaly that so vast a proportion of mankind should thus be permitted to perish in the very prime of life; and are willing to believe that diligent research and extended means of investigation, will yet one day be rewarded by the discovery of the manner of limiting the ravages of this devastating plague. M. Louis believes that future inquiry can only be pursued with probable chance of success, by means of the *association* of a great number of well-qualified inquirers, for the purpose of observing and recording upon an extensive scale all the phenomena of the disease for a considerable space of time, as they may occur in individuals placed under every variety of circumstances. Could a record of this kind be obtained, that it would become a vast aid in improving our knowledge of the etiology and treatment of the disease cannot be doubted: but there are so many obstacles to the forming of one with sufficient accuracy to be depended upon, that we fear it must be placed among the large assemblage of things which are rather desired than hoped for.

In the mean time individual observers are by no means idle, and although the productions they send forth are too often the result of a crude generalization, a misapprehension of fact, or a desire to obtain popularity and its consequences by promising to an ignorant and anxious public that which in the present state of science is impossible; yet enough valuable matter is continually put forth in independent publications, or the British and Foreign periodicals, to shew that a different order of minds has also deeply engaged in the contest with this dread enemy, and thus to stimulate co-operation and prevent despondency.

Dr. Evans, to whose work we shall chiefly confine ourselves in this article, believes that in the amended pathology, which he presents in his "Lectures," we may obtain a rational indication of treatment; and he is by no means disposed to regard the disease so necessarily, or so usually, fatal as do the majority of medical men. We have, of course, no intention of presenting an analysis of so well known a work as that of M.

Louis, but it will be desirable to advert to some of the new matter he has introduced into the present edition. The view this author takes of the probabilities of cure is far less sanguine than that of Dr. Evans, and, although we fear it is the more correct of the two, we cannot but subscribe to the justice of the criticism delivered by the latter upon this celebrated work. He says—

"I have found it necessary to differ in many things from the modern Parisian school of stethoscopists. In M. Fournet's book, there appears to me to be many old observations put forward with an imposing air of novelty, and many new assertions destitute of foundation. M. Louis' work on Phthisis is of a higher order: admirable on account of its careful pathological descriptions, most useful as a repertory of elaborately-drawn cases; yet, I must consider its etiology illogical, its diagnosis rather meagre, and its treatment inadequate."

### PATHOLOGY OF PHTHISIS.

Dr. Evans believes that attention has been unduly directed to the nature, detection, and removal of *tubercle*, as if it were the *cause* of the various symptoms, whereas it is but the result of a peculiar form of inflammatory action, occurring in persons possessing the *phthisical predisposition*. To the examination of this predisposition, as the first stage in the production of phthisis, he attaches great importance; and, after alluding to various circumstances which may give rise to it—as hereditariness, exposure to cold and damp, absence of light, the debility caused by various diseases, &c.—he thus speaks of its nature.

"From a careful consideration of the causes which tend to predispose to the development of tubercle, I think you are justified in coming to the conclusion that this predisposition consists in a deficiency of that manifestation of vital force whereby the tissues are enabled to grow at the expense of the circulating fluid. This deficiency may be congenital, or it may be the result of external circumstances: it may be confined to an organ, or it may implicate the whole system. But we are led by strict induction to believe that when this predisposition exists, the slightest local inflammation is liable to terminate in that peculiar variety of fibrinous secretion intermediate between lymph and pus, to which has been given the name of tubercle.

"Comparative anatomy and embryology prove that the development of muscular tissue is the product of an action of growth, of a higher order than that which gives rise to cellular and nervous tissues, it consequently follows that in a general arrest of development in the organism, the muscular tissue should suffer first, and in the causes which tend to produce atrophy, this tissue should first present a deficiency of nutrition. Therefore it is, that in the predisposition to tubercle, we find a want of proportion between the red and white tissues, the latter are present in excess, and it has been supposed that an excessive development of the white tissues predisposed to tubercle. But from what we have seen you can evidently understand that it is not an hypertrophy of the white tissues, which constitutes the predisposition: but that the same causes that predispose to tubercle, produce likewise atrophy of the red tissues.

"You may perceive how well the locality which tubercle generally occupies, corresponds with the two-fold method of its production; we might be led by pure calculation to conclude that the lungs, of all organs in the body, ought to be most liable to inflammatory affections, subject, as they are to every atmospherical change, and kept in never ceasing activity; and then, on the other hand, the upper lobes are those in which nutrition is least active, and the left side, again, is in all the higher animals, the least developed." 15.

In the Appendix to his work the author examines, at considerable length, the foundations upon which M. Louis rests his statement, that *inflammatory action is not the fore-runner of phthisis.* Upon the observation by this eminent pathologist, that pneumonia, pleurisy, and bronchial inflammation, do not cause the deposition of tubercle, Dr. Evans observes—

"M. Louis considers, that it is impossible to maintain that inflammation is a powerful or common cause of phthisis. But in his treatment of this subject, he seems to me to have fallen into the sophism which logicians call *ignoratio elenchi;* that is, he has proved a thing which has no necessary connexion with, and, therefore, does not determine, the question. The question is, are tubercles a result of *an* inflammation? M. Louis proves, that plastic or suppurative inflammations do not give rise to the deposition of tubercles; and therefore he draws the conclusion, that tubercles do not arise from *an* inflammation. If inflammation was a thing capable of an exact definition, having always the same anatomical appearances, accompanied by the same vital aberrations, and characterized by the same alterations of functions, this conclusion might be justified. But pathologists know that this word is, on the contrary, very vague in its interpretation; is intended to express extremely varied combinations of lesions; and differs very much in its results under various circumstances. Sometimes inflammation produces softening, at others induration; sometimes it causes secretion of pus, at others coagulable lymph; nay, mere congestions from atony or venous obstruction, have been called asthenic or passive inflammations. If M. Louis succeed, therefore, in proving that ordinary pneumonia, pleurisy, or bronchitis, terminate in the majority of instances, without the development of tubercles, we may admit the truth of his deductions, and yet remain as much as ever in the dark, as to whether tubercles are a result of *an* inflammation." 167.

The author next enters into a critical examination of the cases of Acute and Latent Phthisis cited by Louis, as proving that tubercle originates independently of inflammation, but from which he draws totally opposite conclusions.

In the same part of his work, Dr. Evans opposes himself to the generally received opinion of the *identity of the tuberculous cachexia and scrofula.* After quoting Sir James Clark's description of the tuberculous diathesis, and complaining of its vagueness, he adds—

"The fact is that Sir James was partly influenced by experience, and partly by prejudice, in penning the above description. Experience warned him that he had seen Phthisis attacking every variety of constitution; while prejudice suggested to him, that struma being, according to common opinion, identical with tubercular disease, he should mix its acknowledged phenomena as elements of the picture. But is there any sufficient evidence that scrofula is identical with the phthisical diathesis? I think not, and am inclined to regard these states of the constitution as totally independent of each other, for the following reasons. 1st. I see every day numerous examples of phlyctenular ophthalmia, prurigo, ricketts, &c., diseases confessedly peculiar to scrofulous children. I am frequently called upon to prescribe for lymphatic-looking infants, with tumid upper lips, dilated pupils, swollen bellies, and enlarged cervical glands: but upon inquiring as to the diseases to which their parents and other relations have been liable, I do not find that consumption or decline is mentioned more frequently than among any other class of cases. I have known many large families, the members of which, have been all more or less subject to scrofula, in one form or another, and none of them had ever got phthisis. 2nd. Upon inquiring into the early history of numerous phthisical patients, I have remarkably seldom met with persons who at any time presented the characteristics of struma. Nay, in one remarkable instance, when, out of a family of eighteen members, fourteen died of phthisis, not one ever presented a symptom of scrofula, unless acute hydrocephalus in one child could be considered such." 189.

The cause of *Emaciation* in phthisis is thus stated.

"We have already seen that the predisposition to phthisis consists in a diminution of the force of growth, of that vital attraction whereby the tissues draw from the circulating fluid the materials of their nutrition. It is not difficult to understand, that a diminished power of growing is equivalent to an excess of waste, in producing atrophy of the tissues. If this view be correct, emaciation is one of the essential elements of the disease. It is not the result of tubercle, but is produced by the causes which predispose to tubercle; and if this be true, we should be led to expect that not unfrequently a general atrophy should precede any local evidence of the deposition of tubercle." 21.

In respect to the influence of *Hæmoptysis* in producing phthisis, Dr. Evans is again at issue with M. Louis. The latter author observes:

"Hæmoptysis was long considered an exciting cause of phthisis; and M. Fournet, in an extensive work recently published, adopts the obsolete notion. But it is impossible to discover the foundation upon which this writer bases it; for no proposition is at the present day more satisfactorily proved, in the opinion of all accurate observers, than the extreme rarity of hæmoptysis of any amount, unless as a dependence upon tubercles; so that, admitting *argumenti gratia* that attacks of hæmoptysis of this kind, are sometimes the exciting cause of a deposition of tubercles, the fact could not be proved. It is impossible, then, in the existing state of things to regard hæmoptysis, either of considerable or trifling amount, as a cause of tubercles." 505.

Dr. Evans believes, on the contrary, that the profuse hæmoptyses, which are sometimes observed in young people, may give rise to the phthisical predisposition, and thus operate as the cause of tubercle. He says:—

"The question is one of vast practical importance. It involves the consideration of whether we are to put into operation every known means of arresting hæmorrhage, cheered with the hope of being able to ward off the tubercular deposition; or whether we are to feel depressed with the conviction, that the seeds of decay have been already sown. M. Louis' argument comes to this, that because in the majority of cases of profuse hæmoptysis, the patients have ultimately become phthisical, we are therefore to consider tubercles to have been the cause of the hæmorrhage; although it may have occurred in a person previously healthy, and who had not as yet presented either rational symptoms or physical signs of pulmonary disease."

"M. Louis himself acknowledges, that this is frequently the case. He says [p. 167,] 'Either in its severe or slight form, hæmoptysis sometimes occurred a variable time before the appearance of the cough or expectoration. Such was the case with 12 of my patients (out of 57), 8 of whom had had severe hæmoptysis. This form set in still more frequently than the other, in course of, or at the commencement of the first period of the disease, in the proportion of 9:7. Spitting of blood occurred but rarely towards the close of life, when the patient's feebleness had reached the maximum.' With these observations I perfectly agree; and I cannot but thank M. Louis for the candour and truthfulness of his observations, at the same time that I am often compelled to differ from his conclusions." 193.

The rarity of *hæmorrhage at an advanced stage of phthisis* depends, as shown by Lænnec, upon obliteration of the vessels in the neighbourhood of cavities. Of the circulation in the tuberculated lung, M. Louis observes:

" Since the publication of the first edition of this work, the morbid anatomy of the tuberculized lung has made unquestionable progress, more particularly in respect of its vascular system. It was, no doubt, well known fifteen years past, that the ramifications of the pulmonary artery penetrate neither into tubercles, properly so called, nor into gray semi-transparent granulations: but our knowledge on this subject has since then advanced. It is, in truth, now established by the inquiries of M. Schroeder Van der Kolk, and more especially those of M. Guillot, that the branches of the pulmonary artery stop short at a certain distance $1\frac{1}{2}$, 2, or $2\frac{1}{2}$ lines from tubercles or gray granulations; and the more these adventitious productions increase in size, the further do the divisions of the artery stop from their perimeter. To such a degree is this true, that when tubercles are of large size, or have given place to cavities, they may be surrounded by a sort of involucrum, ten lines broad, into which no ramification of the artery makes its way. M. Guillot's injections appear to me to render doubt upon this point inadmissible."

M. Guillot's remarks also prove that, after a short period, small vessels are observed to be developed in this involucrum, which, after remaining awhile unconnected with the rest of the vascular system, communicate with the bronchial arteries, or, through the medium of the false membranes so commonly found on the pleural surfaces, with the parietes of the chest. In proportion as the tubercles augment, and soften, the branches of the pulmonary artery become replaced with this adventitious vascular system. Thus accomplishing " the great transformation of the circulation, one of the most remarkable phenomena attending the evolution of phthisis." The new vessels, which become exceedingly numerous, stop short around tubercles without penetrating their substance. They penetrate however into the prominences of cavities, and ramify upon the bands so frequently found stretching between their parietes.

" Hence, observes M. Guillot, it is not only the highly vascular web surrounding cavities, with its new circulation, that constitutes a striking feature in the anatomy of those excavations,—but further, the terminal tufts which bring the arterial blood, derived from the aortic circulation, into contact with the atmospheric air. The question next naturally arises, how the aortic blood, thus distributed through tuberculous lungs, returns to the heart. The fact ascertained by M. G. that the substance of injections thrown into the aorta is found in the pulmonary, bronchial, and azygos veins, supplies a satisfactory answer to the query. He observes that among the effects of this double current, there must arise a change in the nature of the blood of phthisical subjects, and a special modification of the organism generally,—that in proportion as the tuberculous affection makes progress, so do the lungs, in direct opposition to ordinary laws, acquire increased capacity for arterial blood, and lose it for venous." 30.

*Lesions of the Bronchial Glands in Phthisis.*—M. Louis enters into some particulars respecting these in the present edition.

" Although I scarcely made any mention of the bronchial glands in my former edition, these bodies very frequently undergo tuberculous transformation—not only in children, in whom all accurate observers coincide in regarding the transformation of these organs as even more frequent than that of the lungs, but also after the age of 15 in those of adult years. Since 1825 I have examined the bronchial glands with attention in 70 phthisical adults, and found them tuberculous in twenty (?) to various amounts, or in about one half the cases; whereas, in the same number of persons, the mesenteric glands were tuberculized in 23 cases, and the cervical in 21.

"The bronchial glands, when tuberculized in the adult, are of more or less considerable size, though smaller than in infancy, at which period compression of the trachea and bronchi is a far from rare effect of their enlargement. When thus increased in size, the bronchial glands, unlike those of other parts of the body, exhibit no red discoloration in the spots not filled with tuberculous matter; far from this, they are of greyish and blackish colour, and generally speaking of firm consistence, and in some cases contain only a hard matter, like cretaceous, stony, or osseous substance. The glands thus affected are sometimes, as noticed by M. Andral, of small size, and as it were shrunken: and they are commonly found in this state, according to the same observer, in individuals whose lungs also present appearances of an analogous character, or an incipient condition of cicatrization,—circumstances favouring the belief that they themselves are in the progress of cure. The course of the glandular affection is very variable, and occasionally extremely rapid. Rapidity of course is proved by the discovery of the traces of the affection in individuals, cut off by phthisis in less than two months; for, as we shall hereafter see, the notion that the bronchial disease can have originated—at least in the adult—before that of the lungs, is inadmissible."

Of 26 cases occurring in children, mentioned by MM. Rilliet and Barthez, in 18 the tuberculous cyst communicated with the bronchi or parenchyma of the lung—the communication with the bronchi occurring 15 times, that with the substance of the lung three times.

The following is an abstract of M. Louis' "Summary" of the Pathological Anatomy, founded upon his observations both prior and subsequent to his first Edition.

Recent inflammation of the *lungs* or *pleura* co-existed with tubercles or cavities in $\frac{1}{10}$ of the cases. Ulcerations of the *trachea* in somewhat less than $\frac{1}{3}$, and a changed condition of its mucous membrane in $\frac{1}{5}$. *Pericardium* contained fluid in $\frac{1}{10}$, and exhibited traces of inflammation in several others. The *heart* frequently soft: the *aorta* red in most young persons, and altered in structure in those above 40. *Stomach* very much distended, and its mucous membrane affected in $\frac{1}{12}$, it being softened and attenuated, or softened and very red in $\frac{1}{5}$: ulcerated or mammillated in many. It was quite healthy in but $\frac{1}{5}$. In $\frac{5}{6}$ of the bodies examined, ulcers of the small *intestine* were found, and nearly as frequently in the large. The mucous membrane of the canal very soft in one-half, and healthy from one end to the other in only three cases. Transformation of *mesenteric glands* occurred in $\frac{1}{4}$ of the cases. The *liver* had undergone fatty transformation in $\frac{1}{3}$, and the *spleen* and *kidneys* tuberculous change in about $\frac{1}{6}$. *Prostate gland* transformed in several cases, and the superficial stratum of the interior of the uterus converted into tuberculous matter in three cases. Effusion into the *abdomen* was found in $\frac{1}{4}$, and a small quantity of pus in the pelvis in four cases. *Arachnoid* very frequently thickened and granular. *Pia-mater* infiltrated, and *ventricles* distended in $\frac{3}{4}$ of the cases. The *brain* injected in $\frac{1}{7}$, and its consistence diminished in $\frac{1}{20}$ of the cases. In the same number partial palsy, softening, and, in a few, tubercles, existed.

"The period of origin of these various anatomical changes varied materially; some of them—pneumonia, pleurisy, softening and reddening of mucous membrane of the fundus of the stomach, pulpy softening of that of the colon, acute peritonitis, arachnitis, partial pulpy softening of the brain—set in at the close of life. The majority of them were inflammatory—a circumstance showing that feebleness was less an obstacle to, than a condition promoting, the development of inflammation. The others originated at some more or less remote period from the fatal event, sometimes at the very outset of the disease; such were the vast intestinal ulcerations in some cases, the tuberculous granulations of the meninges, and the chronic peritonitis in others.

"These lesions were, in a certain point of view, of two kinds; some of them appeared proper to phthisis, others were quite independent of its influence, and existed in different degrees in chronic diseases of all kinds. Among the former are ulcerations of the larynx, and more especially those of the trachea and epiglottis, ulcerations of the small intestines, and fatty disease of the liver. So true is this, that the detection of an ulceration in one of the organs just named, &c. might, independently of all further investigation, be considered as the certain announcement of the individual presenting it having died phthisical.

"Another lesion proper to phthisical subjects was *tubercle,* or the grey semi-transparent granulation, or the grey matter in masses. Wherever these products presented themselves, *I have never observed them in any viscus, without their being likewise present in the lungs.* I speak, be it remembered, of individuals who have passed their 15th year. Whence the apparent conclusion that their existence in the lungs forms a necessary condition for their development in other parts."

M. Louis, after observing that when tubercle was co-existent in other organs, as well as in the lungs, it was always found (with one exception) more advanced in these latter organs; alludes to the only exceptions to the law before stated he is aware of.

"However, as my object is not to support any given opinion, as such, rather than another, I am desirous of adding that I have met with an exception to the general law in question. The individual furnishing it died of typhoid fever. There were no tubercles in the lungs, and yet there was a small quantity of tuberculous matter in one of the mesenteric glands. I have mèt with another exceptional case since the publication of the former edition. A third has been recorded in a contemporary journal. But these infinitely rare exceptions do no more than throw into stronger relief the importance, and almost universitality of the law." 154.

We may conclude this section of the subject with quoting the summary of the opinions held by Dr. Evans, as stated in certain "Pathological Propositions."

"1. Phthisis is a disease characterized by a deficient force of growth, together with symptoms both local and general of active pulmonary congestion. 2. The preponderance of white tissues in this disease is due to a diminished force of growth, whereby the tissues generally, but the red in particular, are rendered incapable of attracting from the blood their normal quantity of aliment, and by which their power of resisting decomposing influences of external agencies is diminished. 3. The diminution of the force of growth depends upon abstraction of natural stimuli and aliment: for example, want of heat, light, oxygen in the blood, &c., and the food being insufficient and innutrious. 4. The active pulmonary congestion depends upon the application of stimuli too violent and too prolonged, and may display itself either in the form of bronchitis, hæmoptysis, or pneumonia. 5. The symptoms of active pulmonary congestion in this case, are hectic fever, hæmoptysis, catarrh, cough, altered voice, together with derangement of the digestive and uterine functions. 6. The pathological appearances of the active pulmonary congestion are those of bronchitis, pulmonary apoplexy, or of pneumonia, in the stage of engorgement. 7. The same causes which produce the symptoms of phthisis, are likewise apt to produce the secretion of what is called tubercle, an albuminous substance, intermediate between coagulable lymph and pus. 8. The parts of organs that have secreted tubercle, are subsequently disposed to ulcerate and suppurate, and the tubercle, at the same time, to soften in part, into a fluid similar to pus. 9. Abscesses formed by the softening of tubercles, and the ulceration and suppuration of surrounding parts, are subject to the ordinary law of abscess—viz. burrowing to and bursting from the surface presenting the least resistance, following the least organized track in their fistulous course, cicatrizing by the means of a lining membrane, &c. 10. Masses of tubercle and tuberculous cavities are generally surrounded with indurated lungy substance, of a black, yellowish, or greyish colour. 11. In proportion to the amount of this induration will be the signs of impeded circulation, namely, dilatation of the right cavities of the heart, and enlargement of the extremities of the fingers. 12. The existence of tubercles is not signalized by symptoms, nor their absence a cause of amelioration in disease. 13. The presence of tubercles never causes inflammation in the surrounding tissues. 14. The state of emaciation being a direct consequence of diminished force of growth; and this latter being the predisposing cause of phthisis, we ought to expect emaciation, or something analogous to it, to precede in general the local signs of phthisis. 15. The lesions in phthisis most important to be kept in mind, are the deficient force of nutrition, and the local pulmonary irritation; and the symptoms of this disease, namely, the emaciation, hæmoptysis, hectic fever, cough, alteration of voice, loss of appetite, thirst, constipation, diarrhœa, amenorrhœa, &c., are all, more or less, the consequences of these lesions. 16. Hæmoptysis, when very profuse, may be the cause of the diminished growth, and pulmonary irritation of phthisis. 17. Subacute gastritis may predispose to phthisis. 18. Excessive discharges, as diarrhœa, menorrhagia, &c., may produce diminished force of growth, and thus predispose to phthisis. 19. The suppression of menstruation in a person predisposed, may produce active pulmonary congestion, and thus give rise to phthisis." 32.

### SEMEIOLOGY.

M. Louis presents us with two tables of the *duration* of the disease, derived from the observation of 307 cases. The results are as follow. Three died during the 1st month: between the the 1st and 3rd month 16: from the 3rd to the 6th month inclusive 79: from the 6½th to the 12th month inclusive 100: from the 12½th to the 24th month inclusive 64. From the beginning of the 3rd year to a period extending in some cases over very many years, 42 cases.

He calculates the mortality produced by phthisis, compared with that caused by other diseases, as nearly 1 : 2; for of 358 subjects expiring during 3½ years, in M. Chomel's wards, 123 died of phthisis, and 235 of other affections: and if to the former number there be added 40 others, who, though cut off by other diseases, had tubercles or cavities in the lungs, we find that nearly one-half of these 358 were phthisical at the time of death.

We may notice some of this author's new observations upon Chronic Peritonitis and Meningitis, in connection with Phthisis.

*Chronic Peritonitis.*—The symptoms are generally few and sometimes so slight as to pass unnoticed, but when observed are usually sufficient for diagnosis. The affection comes on at a variable period in the progress of phthisis, sometimes at its very commencement; and is first denoted by tumefaction and slight pain of the belly, fluctuation or tympanitis, more or less considerable, being after a while detected. The fluctuation disappears, but the tympanitis continues. " In cases where it occurs at the outset, without appreciable effusion, the tympanitis diminishes after a certain time, and then the tense resistance of the abdomen becomes more marked, the form of the convolutions of the intestines becomes visible externally, the abdomen appears nodulated, and is extremely elastic, even when the muscles of the parietes are in a state of complete relaxation." Nausea and vomiting are rare, except towards the close, when acute peritonitis supervenes. Where this latter does not occur the patient still suffers much, and the condition of the abdomen is that which most distresses him. In other cases, in which precisely the same anatomical changes have occurred, the patient suffers no pain whatever. In some very chronic cases, the symptoms of peritonitis eventually disappear, the serum being very rapidly absorbed. These symptoms, though few, when associated, are of great diagnostic value; and even have led to the secondary diagnosis of pulmonary tubercles themselves, in cases which gave no index of these from general symptoms, or auscultation.

M. Louis thus sums them up:—

" The existence of chronic peritonitis might be regarded as matter of certainty in any individual who had, within a variable space of time, and in the order in which they are mentioned, experienced the following symptoms.

" 1. Abdominal pain, commonly general and not severe, though very troublesome, without diarrhœa. 2. Increase of size and of sonorousness of the abdomen: which soon after becomes the seat of obvious fluctuation; in cases where there neither exists, nor has existed, any symptom of organic disease of the abdominal viscera, of the liver more especially, of the kidneys, or of the heart. 3. The more or less rapid disappearance of the effusion, after which the abdomen, slightly and generally tympanitic, exhibits on its surface the outlines of the intestines, distended, in consequence of the difficulty with which the matters circulating through them make their way onwards.

" And all this is accompanied with weakness which is not explicable by the state of the lungs, nor by that of the excretions, which are not by any means unnaturally abundant." 246.

*Tuberculous Meningitis.*—The symptoms of this affection, first studied in children, have often been observed by M. Louis in adults, since his former edition, and he confirms the accuracy of its description as given by M. Lediberder. The disease may arise at various periods of the phthisis, and manifests itself at first by severe, continued, headache, the face becoming alternately red and pale, and the intellectual faculties early failing. The cephalalgia is associated with repeated vomiting from the first, and the combination of these two symptoms in a phthisical patient is a very strong indication of tubercles in the meninges. The headache continues intense with exacerbations from 3 to 12 days; the countenance becomes almost idiotic, and the pupils, at first contracted, afterwards dilate. The severe suffering is replaced by a quiet delirium, with intervals of coma; partial paralysis follows, the vomiting ceasing with the headache. The dyspnœa becomes much diminished, as does the fever, or even ceases, even although large cavities exist; but, towards the close of the affection, both return with increased violence. The duration of the disease is seldom more than 15 or less than 8 days, and intermittence in its progress is of rare occurrence.

The researches of Dr. Gerhard of Philadelphia, and of M. Rufz, show that the same symptoms are observed under the same circumstances in children. Indeed, from these it is seen that the meningitis, so commonly regarded as essential in children, is almost always due to tuberculous disease of the pia mater, and consequently is one of the effects of phthisis.

"The study of this species of meningitis is the more worthy of engaging the attention of the pathologist, from the fact that this affection, like chronic peritonitis, often attacks subjects in whom the tuberculous affection of the lungs has not yet made great advance—in other words, subjects who might have survived a long time had not the meninges become implicated in the disease. Thus the 3 individuals, whose cases have just been narrated, had no more advanced disease than tuberculous and grey granulations in the lungs; and of 13 others, children or adults, whose cases I have analysed with this point in view, 2 only had a small cavity in one of the lungs, and 3 some inconsiderable ulcerations in the small intestine.

"We should, however, deceive ourselves, did we imagine, guided by the evidence of the preceding cases, that the diagnosis of tuberculous meningitis is always so easy a matter, and that it is impossible to confound it with any other affection. The following case shows that, in the adult, as well as in the child, the diagnosis of the disease in question may, in some cases, be extremely difficult." 301.

A case is related which put on all the characters of typhoid fever.

### DIAGNOSIS.

Dr. Evans enters minutely into the subject of the *diagnosis* of the different stages of the disease to which he believes attention should be directed. He considers its ordinary division into only two stages (the period prior to and that subsequent to the softening of tubercle), as quite insufficient for any practical purpose. "I have divided the disease into several stages, corresponding to what I believe to be the progress of the lesions; and I have attempted to point out, with the nearest possible approach to accuracy, the signs whereby those stages can be distinguished. The importance of this distinction is becoming every day more apparent to my mind; and I look on it as having been, in many instances, the cause of a fortunate issue to treatment."

He first directs attention to the signs of the *predisposition* resulting from the "diminished force of growth" already alluded to, in which he believes it to consist; and points out that the deposition of *fat* is by no means inconsistent with this theory.

"We have seen that the deficient force of growth is the cause of the emaciation in phthisis, and that it affects the red tissues more than the white. The waste of the tissues, which may be considered as remaining unchanged in this case, gives rise to the different secretions. Some of these secretions are thrown off from the system, others are retained. Among the latter is fat, which, if the oxidation be deficient, may remain in the system, but if enough oxygen be supplied this will also burn away, forming carbonic acid and water. You are aware that sedentary habits, by diminishing respiration, promote the deposition of fat, whilst exercise, by increasing the respiratory process, causes its disappearance from the system; so that, you perceive, there is nothing contradictory in there being at one and the same time a wasting of the muscles and an increase of fat —the first proceeds from a deficient force of growth; the second from too small a supply of oxygen."

Whether such deposition of fat do take place or not in phthisical predisposition depends upon the absence or presence of febrile action; for where this is present, as is usually the case, the augmented quantity of oxygen carried to the tissues, by reason of the accelerated respiration, hastens their decomposition, and prevents the deposition of fat.

"If an individual is observed to become weak and debilitated in the performance of voluntary motions—no matter whether the change takes place slowly or rapidly—and if this deterioration of strength be accompanied by an evident increase of fat, such an individual is in a condition of predisposition to phthisical disease. Again, if an individual be perceived to emaciate sensibly, and if at the same time the pulse be frequent and the respiration hurried, without any evidence of local disease existing whereby these feverish symptoms may be accounted for, this person is equally in a state wherein the slightest irritation occurring in the lungs will suffice to originate pulmonary consumption."

The author next considers the means of diagnosing *pulmonary irritation*, which condition he represents as arising from the application of injurious stimuli, and to be the prelude of, and yet very distinct from, inflammatory action. "When I speak of a stage of irritation, I mean that state in which the tissues are firmer and more dense than natural, more susceptible of stimulation, containing less blood, and deficient in their secretions: while I mean by acute inflammation, a state wherein the part is also more susceptible of stimulation, but swollen with blood, and secreting in an increased proportion."

The evidence of the existence of this pulmonary irritation is the exaggeration of the normal respiratory murmur, termed by Laennec *puerile respiration*. All observers, except Dr. Stokes, have considered this as a sign not of disease in the part where it occurs, but merely as a vicarious increase of respiration to compensate for an imperviousness of some other part of the lung. It is, however, a substantive sign to be heard prior to the occurrence of pneumonia, and at the marginal extent of its prevalence, when existing. Dr. Evans is at issue with M. Fournet in respect to the augmentation of the *expiratory* murmur, during puerile respiration. In the fever so frequently attendant upon emaciation, the respiratory murmur becomes *universally* exaggerated; but this does not result from the presence of pulmonary irritation, but from the predominance of nervous excitement —denoting however the predisposition to pulmonary consumption. A *localized puerility*, without evidence of other disease in the lungs, to which it may be supplementary, is strong evidence of pulmonary irritation; and, still more so if it be found in the upper lobes, and accompanied by signs of predisposition to phthisis.

As this puerility of respiration is a sign of irritation of the parenchyma, so is *shortening of the duration of the inspiratory murmur* the sign of *bronchial* irritation preceding the deposition of tubercle.

"It is very probable that, in the majority of cases, there is both bronchial and parenchymatous irritation: but in such circumstances the sign of bronchial irritation would alone present itself, the mucous membrane first perceiving the contact of the air. If, however, we imagine the efforts made by the lung to produce both these effects to be equal, we would then, in fact, have no evidence of disease at all; for, in exaggerated respiration, the inspiration is prolonged, in bronchial irritation it is shortened, so that the two phenomena would exactly neutralize each other. If we suppose the exertion to perform puerile respiration, to extend the struggle made by the bronchi against the entrance of air, we would then have respiration performed by several distinct jerks, which might be either few, distinct, and audible, constituting what is called an interrupted or *entrecoupée* respiration; or very numerous and indistinct, the dry crackling, ronchus of Fournet."

Irritation existing for a certain, variable period is followed by *inflammation;* but the inflammation occurring in those predisposed to phthisis differs from ordinary pneumonia in its pathology. "It is not serum or unaltered liquor sanguinis that is secreted, but tubercles, and this not in masses infiltrating whole lobules or lobes, but in numerous isolated points. The signs of this form of inflammation are, therefore, as peculiar as the morbid anatomy." They are thus delivered in brief:

"Comparative dullness on percussion, feebleness of respiration, the inspiration short from the obstruction to the entrance of the air, the expiration louder and longer from the lung becoming a better conductor of sound, an exceedingly minute crepitus, scarcely audible except upon a forced inspiration, the respiration gradually acquiring a rough, hard, tubular, character, at first appearing in the expiration. These are the signs by which you are enabled to recognize the supervention of inflammation in the air-cells and parenchyma, when it occurs during the phthisical diathesis." 66.

A consequence of the persistence of inflammation in a lung, for a certain length of time, in a person phthisically predisposed, is the *production of induration,* which the author believes plays a considerable part in inducing various of the symptoms usually attributed to the tubercles themselves. This induration varies from a dark red or blackish, to a yellowish or greyish colour, and is denoted by *dullness* on percussion and the production of *bronchophony.* A contraction of the lung, which occurs simultaneously, causes a drawing-in of the upper part of the thoracic parietes, and frequently a dislocation of the clavicle from its normal position. This induration, by being an obstacle to the admission of air into the portion of the lung where it prevails, often causes an *emphysema* of other portions, in consequence of the effect of increased respiratory efforts there. By the obstruction to the circulation which it causes, it may induce a dilated condition of the right ventricle, hepatic congestion, and disease, diarrhœa, ascites, anasarca, &c. In the lungs themselves it may give rise to serious complications, as hæmoptysis, and aggravated dyspnœa. Any cause of solidification of the pulmonary tissue may produce these effects; and some "ascribe them to solidification from masses of tubercles; but I cannot conceive how any one can do so, who is much in the habit of examining lungs after death: for nothing can be more rare than to find masses of tubercles sufficient to produce these effects."

We need not pursue the author's observations upon the diagnosis of the *softened tubercle,* the production of *pneumothorax,* and of the various descriptions of *cavities*, as these do not materially differ from those found in other works. His summary of the appearances of the *sputa* may, however, be extracted :—

"The sputa expectorated at different periods in phthisis, varies according to the lesions existing in the several stages. During the stage of irritation, all secretion is suspended, and the cough is consequently dry. If the stage of inflammation consist in a bronchitis, the sputa will vary, according to the nature of the catarrh. Very rarely it is the crude bronchitic sputa, transparent, coagulable by heat, and very albuminous: more frequently it is the concocted sputa, opaque, in defined clots with ragged edges, rendered lighter than water by the bubbles of air mixed with it, but actually specifically heavier, consisting of very little albumen, principally of the spherical granulated globules of mucus, suspended in a viscid fluid, often alkaline in its re-action. This kind of sputa is sometimes, again, mixed with pus, derived from a true suppurative secretion of the mucous passages, more albuminous than the mere mucus, and yielding an oil to æther when agitated with it. This variety of sputa is a sign of sub-acute, or even asthenic inflammation of the lining membrane. A still more common variety of sputa in phthisis, especially in extreme cachexia, is the pituitary. This is expectorated in enormous quantities, has the appearance of the serum of whey, is white and frothy on its surface; the microscope detects in it numerous flakes of epithelium, but few or no mucous globules, and it is scarcely, if at all, albuminous: it has a very salt taste. When this kind of sputa is present, emphysema will be often found to come on. After the establishment of bronchial fistula, with a tuberculous cavity, the sputa generally alters in its character. It now becomes highly albuminous; when examined by the microscope it is found to abound in pus globules and other globules much resembling those of pus, but varying exceedingly in size. These are softened tubercles. White cheesy flakes are also to be observed in it, which sometimes so much abound as to give it a pulpy consistence; at others, these are very scanty. In all the foregoing varieties of sputa, blood globules may occasionally be seen; but the true rusty sputa of pneumonia, is scarcely ever to be recognized in phthisis." 67.

Dr. Evans, in concluding the subject of diagnosis, passes in review the various fallacies to be avoided in the different stages; such as mistaking the enfeebled and bloated condition of the system attendant upon great inactivity of the large intestine for the debility of phthisical predisposition —the shortening of the inspiratory murmur produced by hysteria, intercostal rheumatism, &c., for that of pulmonary irritation—the signs of cured pneumonia or pleurisy for those of induration, &c.;—and concludes with the following estimate :—

" 1. That the symptoms of phthisical predisposition are of greater value in proportion as they are evidently essential. 2. The signs of pulmonary irritation and bronchial inflammation acquire their chief importance from occurring in persons phthisically predisposed. 3. The signs of tubercular parenchymatous inflammation are peculiar, and have a positive diagnostic value, but this is increased by the co-existence of a phthisical predisposition. 4. The signs of induration, as regards the diagnosis of tubercles, are only of value in combination with the rational symptoms of phthisis. 5. The signs of cavities acquire all their importance from the history and progress of the case, in connexion with rational symptoms.

" I may be permitted to add a few observations :—1. As regards *hæmoptysis*, you will often meet with persons who have never spat blood from the commencement to the termination of phthisical disease. On the contrary, hæmoptysis, although profuse, if evidently vicarious, is not necessarily a bad symptom: and it is no uncommon thing for it to prevail, especially during the first months of pregnancy, in certain individuals, in the same way as others will suffer from pyrosis, or profuse salivation. With respect to *purulent expectoration,* it is a symptom of no value taken alone. Dr. Green, of this city, has lately shewn, that, during the absorption of empyema, a very copious purulent expectoration sometimes occurs, without any bad effect. 3. The sign of *diminished vibration of the voice,* as felt by the hand, is really of no value in phthisis; for this vibration is naturally greater under the right clavicle than under the left, and even a considerable degree of induration on the right side, sufficient to produce the most marked dullness on percussion, will be rarely capable of diminishing the vibration enough to cause it to be equal on both sides."

### Prognosis.

The question of the *curability* of phthisis is one of as difficult decision as of vast importance. Judging from the vaunts so frequently made in the present day of the specific powers possessed by so many medicinal agents, we might seem to have arrived at that stage of the inquiry which does not admit of farther doubt being entertained. But, alas! when we come to examine into the history of reputed cures, how often are we met with evidence of want of good faith, and still oftener of accurate knowledge, upon the part of the observer. The well-known uncertainty of the duration of a given case of phthisis, and the numerous instances in which the symptoms have been arrested, or have stopped short, for an extraordinary length of time, open a wide field both for charlatanism, and for self-deception; while numbers of cases which have been set down as cured, were never examples of phthisis at all, and remain mere monuments of the ignorance in diagnosis of those who have had the management of them. Believing this, however, we think it is no less the duty of all who have the opportunity of pursuing the investigation [and who has not?], to enter diligently upon the trial of various modes of cure, even when as empirically suggested as many of those to which we have alluded are; for not only is the possibility of the eventual discovery of the right one not unreasonable, but many incidental advantages may spring from the researches made in quest of it. To the alchemists in their vain search we are indebted for the discovery of many valuable processes and preparations. But, if we mean to maintain the character of sober, honest, and rational inquirers, we shall employ a little more caution, both in assuring ourselves of the presence of the disease to be cured, and in the frequency with which, and duration of time during which, we test our means of relief, before we offer the results to the profession, much less to the *public,* than has of late been the fashion.

M. Louis refers to three cases in which the disease, not having made much advance, became permanently arrested: and he considers that the researches of Laennec and others have proved beyond doubt the possibility of the cure of phthisis. Those of Dr. Rogee, quoted upon this point, may be alluded to. From them it would seem that the cretaceous or calcareous concretions, formed at the apex of the lungs, are always the sequel of cured or transformed tubercle. Now as 51 out of 100 women, opened by him at the Salpêtrière, without selection, presented one or more of these concretions, phthisis appears to be more frequent in its occurrence, and far more frequently disposed to stop short in its career, than was imagined. It is however difficult to suppose these cases could ever have been of a very serious kind, or that large cavities are ever transformed into these little masses. Even the period of life when such anatomical changes commenced is very doubtful, observed as they were in aged subjects.

"It appears from all that precedes, how difficult a matter it is to form a prognosis in phthisis, and into what an abundance of errors we should be drawn, were we to attempt to establish it on the invasion of the affection. On the one hand, a certain amount of severity in the symptoms at the outset does not always prevent the disease from stopping short in its course. On the other hand, we have seen that the affection, after having advanced for a certain number of years with much, nay, extreme slowness—perhaps even after having stopped completely for a season—may suddenly assume all the characters of severity, and cut off its victim in a very short space of time. Perforation of the lung may take place very soon after the invasion of the malady; tubercles may form in the meninges; or chronic inflammation seize on the peritoneum. It is impossible, in the present state of knowledge, to foresee the time at which these symptoms will manifest themselves in individuals destined to experience them, or to prognosticate who will be afflicted with, and who exempt from, their development. There are reasons in abundance to prove the wisdom of caution in prognosis:—while at the same time their general tendency is to display the danger of the affection, and the small hope of prolonging existence, when the symptoms assume a severe character at, or soon after, the period of invasion." 476.

Dr. Evans takes a far more, and, unfortunately, a far too, favorable view of the prognosis of phthisis.

"If, you insist on the discovery of means whereby tubercles may become absorbed, or induration be resolved, or abscesses be made to disappear, and be replaced by normal structure, I confess that I am ignorant of any remedial method, whereby such wonders can be effected. But if you look on a pleurisy as being cured, although the side be contracted and the lung compressed; if an ulcer is considered healed, although an unsightly cicatrix occupy its site; then I promise you, that by pursuing a proper line of treatment, you will be enabled to cure many cases of phthisis in every stage. In many instances of incipient phthisis you may succeed in removing every trace of the disease, and leave your patients in as good health as before they were attacked; and in other more advanced cases, you may at least alter the disease from being a rapid and fatal one, into a malady, troublesome no doubt, and requiring constant care, but not more distressing than the generality of chronic affections. These are strong statements, gentlemen; but I hope to be able to support them by equally powerful evidence."

To this evidence we shall anxiously look for confirmation of so remarkable a statement; but may first advert to M. Louis' observations upon the

ETIOLOGY,

And which he confesses are rather "materials for combating error than establishing truth."

1. *Predisposing Causes.*—(1.) *Age.* The examples of tubercle in the fœtus and early months of extra-uterine life are rare, and M. Guyot opened the bodies of 400 new-born infants without meeting with one. According to the extensive researches of Papavoine, tubercles more usually appear during the first dentition, especially when it is anormal; but still they are far less frequent during the first two years than afterwards, between the ages of four and seven, being the period of greatest prevalence in childhood. The age at which the disease is most prevalent in adults is well known. (2.) *Sex.*—Females are more liable than men. Of 123 cases analysed, 70 were furnished by females, and 57 by males. (?) Out of an equal number of each sex, admitted into the wards for chronic diseases, 40 were cut off with phthisis in the proportion of 25 women to 15 men. Here we cannot but observe upon the *very frequent* typographical errors which so disfigure M. Louis' work in respect to many of the details and totals of his calculations. A strange oversight in one who lays so much stress upon accurate figures as he does, and which might have been obviated in the translation by communicating with him upon the subject. M. Benoiston de Chateaûneuf found, by the Paris hospital registers for 1821-36, that $\frac{1}{35}$ of the males, and $\frac{1}{25}$ of the females died of phthisis. Mr. Farr has drawn attention to this preponderance of females in England also. (3.) *Constitution.*—M. Louis strongly doubts that delicacy and weakness of constitution predispose to this affection. He does not state this as a fact, but reasons from analogy, having observed the course of phthisis to be run as rapidly, or more so, in strong than in weak subjects. No reasoning from such a defective analogy as this can however be admitted. (4.) *Temperament.*—Much is not known of the influence of this; but as the lymphatic is most prevalent in women, and, according to Papavoine, in girls, and as females are more liable to phthisis, this temperament may act as a predisposing cause. (5.) *Hereditariness.*—M. Louis has observed nothing decisive in favour of this; and considers M. Briquet has received evidence of its general prevalence upon far too slight grounds. (6.) *Bad Diet, and Violation of Hygienic Laws.*—Although agreeing with Sir J. Clark, that these influences must tend to generate chronic disease, and phthisis among the rest, M. Louis is by no means disposed to allow, with that talented physician, that they have an especial tendency in generating the latter—at least before he can do so, he must have better evidence than the recital of a few cases, which may be mere coincidencies, presents. If these causes have so much power in generating the affection, they ought to possess that of hastening its outbreak; but in 30 patients carefully examined, 12 of whom had been badly nourished, and 18 had been submitted from infancy to no privation whatever, yet the average age of the development of the disease was precisely the same. (7.) *The Influence of Occupation* is one of the most difficult pathological problems, owing to its complications, and the numerous elements necessary to be taken into account in any analysis. The question is quite *sub judice* at present. (8.) *Clothing.*—M. Louis doubts the malignant influence supposed by most to be exerted by *stays*. Most of his patients were peasants, who had never worn these until after their arrival in Paris, when their growth was complete; and it is to be remembered that phthisis also predominates in the female before as well as after 15, and consequently before she has commenced using this article of dress. (9.) *Climate and Temperature.*—It was formerly generally believed that warm countries, especially those of the South of Europe, were exempt from phthisis; but this is now known not to be the case, and Dr. Journee has shown that tubercles are found as frequently in the inhabitants of the chief towns of Italy as in Paris. M. Louis also observes that the opinion of the influence of abrupt variations of temperature being very conducive to the production of phthisis, and great uniformity a powerful means of warding it off, has no evidence in its favour, and refers to Major Tulloch's Report on the Health of the British Army as militating against it.

2. *Exciting Causes.*—(1.) *The Influence of Pneumonia, Pleurisy, and Bronchitis.*—M. Louis' opinions upon this have been already stated at the beginning of this article, as also upon the question of *Hæmoptysis* being an exciting cause. (2.) Non-*inflammatory excitement of the Lungs.*—The little influence of this is seen in the fact, that of 42 subjects of cardiac disease, only two presented tubercles. (3.) *Marked and prolonged febrile action,* as in *typhoid* fever, may excite the development of tubercle. (4.) *Cold* has long been considered as a powerful exciting cause of the production of tubercle; but, if so, the disease should be especially developed in the Winter months. But of 170 phthisical patients in La Charité, 74 experienced their first symptoms in the warmest, and 76 in the coldest ones; and of 127 patients in Beaujon, 66 contracted the disease in the warmest, and 61 in the coldest months. Women, too, who are more subject to the disease, are better clad than are men in Paris. It is said that cold exerts this effect upon the Quadrumana of the Menagerie in Paris; but then the cows, which are kept in hot, close, stables in that city, are very liable to phthisis also.

### Treatment.

M. Louis passes in brief review "the principal means which have of late years been brought forward, as best calculated to arrest the progress of phthisis."

*Protioduret of Iron* was introduced with great laudation by M. Dupasquier, but neither this or any other preparation of iron has proved useful in the hands of M. Louis. He delivers a caution well worthy of notice at the present time.

"It is true that, in order to obtain accurate conclusions, I took care not to put my phthisical patients on any of these ferruginous preparations till six or eight days after their admission; because daily experience shows that a few days' regulated diet, combined with the use of diluent drinks, will suffice, perfectly unassisted by active treatment of any kind, to produce an improvement in the state of their various functions, to cause decrease of thirst, improvement of appetite, a better appearance of the sputa, greater facility in expectorating, &c. It is perfectly clear that, unless the precaution to which I now refer be taken, a certain improvement, in reality depending upon regimen alone, may be ascribed to the influence of some pharmaceutical preparation, and the observer be thus deceived into most serious errors. It is more than probable, that omission of this precaution accounts, in great measure, for the utterly different views of practitioners concerning the action of medicines."

*Chloride of Sodium* was recommended by M. Latour in doses gradually increased from ʒ½ to ℥¼, and continued for two or three months, but, after a patient trial M. Louis found the remedy to be worthless. Of the *Subcarbonate of Potass*, of large doses of *Sal Ammoniac*, and of *Chloride of Lime* he has had no experience. The inhalation of *Chlorine* has been attended with no success in his hands. The few cases he has tried *Digitalis* in have furnished no satisfactory results, nor has he been more successful with *Prussic Acid*. Of *Iodine* and *Creosote* he knows nothing, and the fame of *Naphtha* had not extended to Paris at the period of his publishing this edition. The scepticism with which most observers regard the loudly vaunted pretensions of new medicines for the cure of phthisis, has a tolerably good justification in the history of the above-mentioned ones. Each upon its introduction has been accompanied with vouchers of having cured consumption, no matter how advanced, and yet have they all, one after the other, sunk into merited oblivion.

M. Louis' chapters upon the prophylactic and palliative treatment of this distressing malady, although containing many useful remarks, are by no means equal to the other portions of his work; and we may now turn to Dr. Evans' "Lectures," in which decisive efforts are recommended, not for the mere retardation of the disease, but for its curative treatment. He strongly protests against the too prevalent custom of treating the symptoms of diseases as they arise, without endeavouring to refer them to the lesions upon which they are dependent; and adds that any benefit which can be expected to accrue from the treatment he recommends, will be only proportionate to the accuracy with which the different stages of the disease are recognized. The lesions he distinguishes as characteristic of phthisis occur in the following sequence: a diminished force of growth and reparation, with its consequent excess of nervous excitability: local pulmonary irritation: local pulmonary inflammation with the secretion of tubercular matter: a state of pulmonary induration: a condition of ulceration and suppuration of the lungy substance. *Tubercles* are only the *result* of phthisis, "they are incapable of producing any of its symptoms, and their presence has very little influence on the progress of the disease." *Their* treatment must never be the object we have in view. "We have no positive means of judging when they are present. When present we know of no treatment for them; and the best method of preventing their deposition, is by checking the local pulmonary inflammation, which is their immediate cause." We will now furnish an abstract of the mode of treatment recommended by Dr. Evans in these various stages.

1. *Stage of Predisposition.*—The diathesis may be congenital, or produced by cold, moisture, want of light and bad food, when change in all these particulars may be followed by amendment. A sea voyage to a mild climate, changing so many of these conditions, has often proved of great efficacy. Then the heart's action and the digestive power may be augmented by the judicious use of tonics and alkalis. When there is embonpoint with a pulse of ordinary frequency, wine and fermented liquors may be given to an extent not to cause fever. But when fever and emaciation are present, they are improper. Such fever, arising from the greater predominance of nervous power, is amenable to proper doses of opium and prussic acid. In some cases the predisposition seems to originate from the presence of sub-acute *gastritis*, which is often very latent, interfering with the due digestion of the food. Here the primary object is the subdual of the gastritis by the avoidance of stimuli and irritating food, the application of leeches, and afterwards blisters or croton-oil ointment, the exhibition of alkalis very much diluted, and small doses of morphia and prussic acid. The phthisical diathesis, in this case, must be combated by the diminution of waste, as by causing the patient to breathe an atmosphere containing little oxygen, either by sending him to a warm climate, or diluting the air he respires with nitrogen or carburetted hydrogen. Mild emollient drinks, as sugar or gum and water, also diminish waste by saving the tissues from the necessity of combination with oxygen. In other cases the predisposition originates in hæmorrhage, especially *hæmoptysis*, which it is therefore very important to check. Repose of body and mind, the avoidance of all stimuli, whether internal or external, as warmth, or the inducing of plethora by too much drink or suppression of secretions, are our principal means of meeting it. The means of diminishing waste are still more limited than in the case in gastritis, as inhalation of gases of any kind would prove very hurtful. The use of gum-water, &c., and the diminution of sympathetic irritation by prussic acid, are all we can advise. It is thus very important to be enabled to relieve hæmoptysis *before* the phthisical predisposition can be established; and this is best accomplished by combating the plethora with venesection or cupping, and abating the activity of the circulation by full doses of digitalis, tobacco, or hydrosulphuret of ammonia. Nauseating medicines, short of causing vomiting, are very useful. The nervous sympathy is best allayed by prussic acid. The local pulmonary irritation, which first caused the hæmorrhage, and may reproduce it, claims particular attention. The application of bladders of ice to the chest, and the administration of large doses of colchicum or turpentine, as contra-stimulants, best effect its removal. The remaining irritation is removed by the exhibition of saline purgatives, and the application of active counter-irritation to the chest. If, after these energetic means, relapses still occur, acetate of lead and large doses of mineral acids should be given; continuing the counter-irritation to the chest, and dry-cupping between the shoulders. The strictest regimen is indispensible. Any other *flux*, allowed to continue, may engender the predisposition. *Excessive secretion* from an organ may induce the diathesis, as in pituitous catarrh and diabetes mellitus. *Diseases of the blood*, in which there is a diminution of some of its elements, as albuminuria, chlorosis, purpura, and scorbutus, are in themselves instances of the phthisical diathesis. *Contagious febrile affections*, as typhus, measles, variola, &c. often give rise to this diathesis, as does the poison of *syphilis*.

2. *Pulmonary Irritation.*—When this becomes established several circumstances act injuriously—such as mechanical exertions of the lungs in singing, talking, &c.—tonics, too nourishing regimen—fever—suppression of fluxes—some of which may be useful in the predisposition, prior to the establishment of the local irritation. Irritation may be induced by direct impression of too powerful a stimulus on the tissue of the lungs, or sympathetically by a distant inflammation, especially chronic laryngitis, metritis, and nephritis. The treatment, in these latter cases, consists in first relieving the distant inflammation, and then the secondary pulmonary irritation. For this last, Dr. Evans recommends the inhalation of watery vapour, the air of the apartment being surcharged with moisture, by means of vessels of water constantly heated by lamps. Counter-irritation must be applied to the chest by the application, with a sponge, two or three times a day, of a liniment composed of *equal parts of turpentine and distilled vinegar*, made into an emulsion with yolk of egg. Small doses of opium or prussic acid are also highly useful. Repose of the respiratory organs, absence of stimuli, and a farinaceous diet are essential. The counter-irritation must be continued after necessity for it has apparently ceased to exist; and, on the subsidence of the irritation, the various modes of combating the predisposition must be resumed.

3. *Stage of Inflammation.*—The treatment of this is the rock upon which so many split. For, if it be attempted to be cured like inflammation existing independently of a diathesis, the cachexia becomes much aggravated; while if it be neglected, the progress of the disease cannot be stayed. The circumstances which relieve the inflammation aggravate the cachexia, and *vice versa*, so that it requires great tact to determine when either class of measures must succeed the other. The inflammation must be first conquered, and for this purpose general bleeding is not admissible, nor even local, carried to a considerable extent. Leeching day after day is a very bad practice. Colchicum and turpentine given in as large doses as can be borne, with long intervals between each, form good contra-stimulants. But the means above all that will be found useful is the *persevering and active employment of Counter-Irritation*: and upon the employment of which the author justly considers himself an authority, having acted for some years as the assistant of the notorious quack JOHN LONG! of whom he says, "After all, I have no hesitation in saying that St. John Long was less of a charlatan than many physicians of the present day, with an alphabet attached to their names; and if his patients placed unbounded confidence in his skill, the reason was, because he actually performed many extraordinary cures." This exaggeration is not to be wondered at on the part of one who stands a confessed accomplice in Long's iniquities, and has only the following lame defence to offer.

"I confess that at the present moment, after twelve years of regular medical study, I do not think the time that I was so strangely engaged assisting St. John Long by any means mis-spent. The almost intuitive knowledge that we gained of the appearance of patients labouring under the disease, and of their symptoms, from the constant communication with such multitudes as were then constantly presenting themselves before us, has been, I have since found, of infinite service to me. Although at the time I was ignorant of everything connected with anatomy and pathology, as any gentleman need wish to be, I soon discovered that St. John Long's treatment was not infallible, and I have reason to believe that he lost great numbers by his total ignorance of diagnosis as regards the particular stages, and of the proper treatment of particular symptoms. Yet, I could not disguise from myself the fact, that numberless cases that presented all the symptoms of confirmed decline perfectly recovered under his care.

"Looking back on the hours which I once spent with St. John Long, and comparing what my feelings then were with what they are at present, I can scarcely believe in my own identity. It is a curious subject for contemplation; the very different aspects which circumstances assume, according to the point of view under which we consider them. I once thought that course an agreeable pastime, which now appears a reckless trifling with human health and life. I once reckoned it as amusing a thing to rob the doctors of their patients as the lawyers of their clients. I need not say that my sentiments are now considerably altered. I did not think there was any thing intrinsically wrong in St. John Long's occupation. When we failed in curing a case, I was sorry; when we succeeded I was glad, but that was all I thought about it; and I am sure those who know me will bear me witness, that, had I conceived there was any thing morally erroneous in his pursuit, I would never have patronized him in it. I believe these to be the sentiments of the majority of persons out of the profession; and this consideration makes me cautious in attributing improper motives to others, although they may be engaged in pursuits that my judgment may condemn."

The smallest share of the commonest sense ought to have taught any one moving in the sphere of a gentleman, "having nothing to do but to amuse myself," that there was something more than "morally erroneous" in joining a fellow, whom he had formerly only known as a painter, in his new avocation of "endeavouring to establish his reputation as an empirical curer of consumption!"

We are willing, however, to allow that this derogatory apprenticeship has furnished the author with useful experience as to the capabilities of counter-irritation, which he states was the sole instrument in the hands of Long. He believes that, by the free use of the liniment, before mentioned, applied all over the chest, after moderate local depletion, an entire cure of tubercular inflammation may be generally wrought. Small doses of opium, or prussic acid, may be given at the same time, and the patient made to breathe a moist atmosphere. After the inflammation is subdued the cachexia is to be met with the means already mentioned, such as a sea voyage to the Mediterranean, careful regimen, and the persistence in a gentle counter-irritation.

4. *Stage of Induration.*—This state is not amenable to direct treatment, but some of its consequences, especially congestion of the lungs and liver, may be usefully treated by *dry-cupping*, and, when hæmoptysis or diarrhœa result, by the use of *astringents;* while the danger of acute inflammation occurring in this condition of parts, may be frequently obviated, by establishing a drain by means of a *seton* applied over the seat of the induration.

5. The stage of *Ulceration and Suppuration* "is signalized by the tendency to new tuberculization, and by the exhaustion following profuse secretion from the walls of the pulmonary cavities." Dry-cupping, seton, narcotics and astringents are here indicated, while distressing cough is relieved by inhaling watery vapour impregnated with sedatives or narcotics. Four cases in which, after all the signs of cavities presenting themselves, the patients regained health, are related: but very few particulars are given.

It may be thought we have dwelt too long upon Dr. Evans' work; but, although we cannot agree with him as to the great extent of the curability of Phthisis, we believe his book likely to prove of great utility in directing attention more distinctly to the different stages of the disease, and the due modifications of treatment in these. Impressed with the hopelessness of the issue of a case of phthisis, practitioners too often abandon all attempts at modifying and retarding its progress. This should not be; for, independently of the not infrequent possibility of a too unfavourable diagnosis having been made, much may often be done to delay a termination which yet must come. Still, we believe the treatment of the affection is invested by Dr. Evans with a deceitful simplicity, and feel certain that he has often confounded simple inflammatory, and non-inflammatory, affections with the true tubercular disease. According to his statement, the management of this terrible malady may be placed in the same category with that of the various other chronic affections. Would that it were so!

Of M. Louis' work we need say nothing farther, generally esteemed as it is for the pathological accuracy and philosophical spirit that characterize it. The sections upon the prevention and treatment of consumption, however, are certainly defective.

Some dissatisfaction has been expressed at the selection of this work for publication by the Sydenham Society; yet it would seem that a body bearing such a title could hardly do better than extend the knowledge of the productions of one of the great Masters of Medical Observation. That a translation of this new edition ought to be furnished from some source is obvious, seeing that it contains the additional experience, all of a confirmatory character, of its talented author during the last twenty years. That its execution could have fallen into better hands than those of Dr. Walshe we do not believe; and we have only to regret the mistaken delicacy which has restrained him from adding notes to it. We hope the operations of this flourishing Society will not deter our medical publishers from entering into honorable rivalry with them. We feel convinced a wide field, in the republication of the works of the best English authors, and translations of those of foreigners, at a rate which must secure a large body of purchasers, is open before them.

In the "Synoptical Account" placed at the head of this article, Dr. Brown gives an account of the success which has attended the application, in cases of Phthisis and Asthma, to the chest of different fluids, and which he supposes is effected upon the principle of Endosmose. He alludes to the testimony afforded by Dr. Hall, in a number of the Lancet of last year, to the great efficacy which spirituous applications, maintained in constant contact with this part, exert in checking the progress of incipient phthisis. The fluids employed by Dr. Brown are of various composition, as diluted Alcohol, diluted Sulphuric Æther, Combinations of Anodynes, Iodine, Astringents, Acidulous and Alkaline Preparations, &c. He applies them just below the clavicle, by means of a piece of hollowed cork, filled with sponge, or of an apparatus manufactured for the purpose by Taylors, 37, Strand. The applications are renewed three or four times a day, and he states their use has been attended with great success. Of this he thus speaks:

"Now several questions will naturally arise in the minds of my readers; and the first of these will be, are these all the cases of Phthisis and Asthma you have had under your care since you hit on this method of treatment; in other words, [since you mention nothing but success as being attendant on your plan, at any rate as far as Phthisis is concerned] do you mean to tell us that with it you have cured or relieved all of Phthisis and Asthma that have come before you since you possessed it? Most certainly not:—a goodly majority, rather above three-fifths, but certainly not all, and as to phthisical cases, they were, with two or three exceptions, in which cavities appear to exist, all in the early stages. Then, did you rely solely on this Endosmotic treatment, or did you accompany it with any other? I relied upon this to effect direct good, but I have always, at the same time, endeavoured to bring about indirect good through the medium of the constitution, by means of tonics, alteratives, change of air, and the other methods usually had recourse to, to improve the constitution."

Many persons will differ from the author as to the relative importance of this means, and the more general treatment he employs at the same time; but few can object to afford the extent of its efficiency a fair trial. As dates are every thing in estimating the power of proposed remedies for consumption, it is as well to observe, that the author's experience of this method had not extended, when he wrote, over a much longer space of time than a year.

